# Expression of the *Acidothermus cellulolyticus* E1 endoglucanase in *Caldicellulosiruptor bescii* enhances its ability to deconstruct crystalline cellulose

**DOI:** 10.1186/s13068-015-0296-x

**Published:** 2015-08-13

**Authors:** Daehwan Chung, Jenna Young, Minseok Cha, Roman Brunecky, Yannick J Bomble, Michael E Himmel, Janet Westpheling

**Affiliations:** Department of Genetics, University of Georgia, Athens, GA USA; National Renewable Energy Laboratory, Biosciences Center, Golden, CO USA; Oak Ridge National Laboratory, The BioEnergy Science Center, Oak Ridge, TN USA

## Abstract

**Background:**

The *Caldicellulosiruptor bescii* genome encodes a potent set of carbohydrate-active enzymes (CAZymes), found primarily as multi-domain enzymes that exhibit high cellulolytic and hemicellulolytic activity on and allow utilization of a broad range of substrates, including plant biomass without conventional pretreatment. CelA, the most abundant cellulase in the *C. bescii* secretome, uniquely combines a GH9 endoglucanase and a GH48 exoglucanase in one protein. The most effective commercial enzyme cocktails used in vitro to pretreat biomass are derived from fungal cellulases (cellobiohydrolases, endoglucanases and a β-d-glucosidases) that act synergistically to release sugars for microbial conversion. The *C. bescii* genome contains six GH5 domains in five different open reading frames. Four exist in multi-domain proteins and two as single catalytic domains. E1 is a GH5 endoglucanase reported to have high specific activity and simple architecture and is active at the growth temperature of *C. bescii*. E1 is an endo-1,4-β-glucanase linked to a family 2 carbohydrate-binding module shown to bind primarily to cellulosic substrates. We tested if the addition of this protein to the *C. bescii* secretome would improve its cellulolytic activity.

**Results:**

In vitro analysis of E1 and CelA shows synergistic interaction. The E1 gene from *Acidothermus cellulolyticus* was cloned and expressed in *C. bescii* under the transcriptional control of the *C. bescii* S-layer promoter, and secretion was directed by the addition of the *C. bescii* CelA signal peptide sequence. The vector was integrated into the *C. bescii* chromosome at a site previously showing no detectable detrimental consequence. Increased activity of the secretome of the strain containing E1 was observed on both carboxymethylcellulose (CMC) and Avicel. Activity against CMC increased on average 10.8 % at 65 °C and 12.6 % at 75 °C. Activity against Avicel increased on average 17.5 % at 65 °C and 16.4 % at 75 °C.

**Conclusions:**

Expression and secretion of E1 in *C. bescii* enhanced the cellulolytic ability of its secretome. These data agree with in vitro evidence that E1 acts synergistically with CelA to digest cellulose and offer the possibility of engineering additional enzymes for improved biomass deconstruction with the knowledge that *C. bescii* can express a gene from *Acidothermus*, and perhaps other heterologous genes, effectively.

**Electronic supplementary material:**

The online version of this article (doi:10.1186/s13068-015-0296-x) contains supplementary material, which is available to authorized users.

## Background

The ability to deconstruct plant biomass without conventional pretreatment is an important attribute for any organism being considered for the consolidated bioprocessing of plant biomass to fuels and chemicals. Existing methods for biomass pretreatment typically rely on physical, chemical and enzymatic hydrolysis, and the cost of enzyme cocktails is a major economic barrier to the use of plant biomass as a substrate [1–4]. The most effective commercial cocktails of enzymes used in vitro to pretreat biomass contain cellobiohydrolase I (CBH I), cellobiohydrolase II (CBH II), β-d-glucosidase and endoglucanase I (EG I) that act synergistically to release sugars for microbial conversion to products [1, 4]. In nature, microbes produce enzymes in structures like the *Clostridium thermocellum* cellulosome [5–8] or free enzymes that act independently, such as those produced by most fungi and cellulolytic bacteria [9]. *Caldicellulosiruptor* species contain a variety of enzymes predicted to be involved in plant biomass deconstruction, and the synergistic activities of these enzymes are likely responsible for their ability to utilize plant biomass without conventional pretreatment [10, 11]. The *C. bescii* genome contains 52 annotated glycoside hydrolases; of these CelA is the most abundant protein in the secretome [12] and the only enzyme to combine both a GH9 and GH48 catalytic domain [13]. Deletion of CelA in the *C. bescii* genome, in fact, resulted in a significant reduction of its ability to deconstruct plant biomass likely because of the reduced exoglucanase activity (GH48 activity) in the mutant [14]. Cellulolytic microorganisms in nature possess high endoglucanase activity conferred by one or two highly active GH9 or GH5 endoglucanases [15, 16]. The *C. bescii* genome contains six GH5 endoglucanases. Three similar GH5 domains are in the same cluster annotated as CbMan5A, ManB, and CbMan5C [17]. Four of the six GH5 domains are present as a part of multi-domain enzymes (one each in Cbes1859 and Cbes1865, and two domains in Cbes1866; Additional file 1: Table S1). Two are present as singular domains and found outside of the cluster (Cbes0234 and Cbes0594; Additional file 1: Table S1). Several of these genes are upregulated on switchgrass [18]: Cbes0234 (GH5 only), 17-fold; Cbes1865 (GH9–CBM–CBM–CBM–GH5), 23-fold; Cbes1866 (GH5–CBM–CBM–CBM–GH5), 7-fold. Thus, most of the GH5 endoglucanases in *C. bescii* are multi-domain proteins and that, given the accepted mode of action of endoglucanases, could potentially limit their contribution when compared to highly active and smaller endoglucanases [19]. For example, biochemical and mutational analyses of Cbes1865 (GH9–CBM–CBM–CBM–GH5) showed that after deletion of the GH9 module, the truncated protein had an increased apparent *K*_cat_ value 2- to 3-fold higher on several mannan substrates, including locust bean gum, guar gum, and konjac glucomannan [20]. The potent GH5 family endo-1,4-β-glucanase from *A. cellulolyticus* 11B, is different from those found in *C. bescii.* E1 is linked to a family 2 CBM instead of a family 3 CBM and contains a single catalytic domain [21, 22]. This structure might allow a versatility prevented by the large, multi-modular enzymes found in abundance in *C. bescii*. For instance, E1 may have more accessibility to the substrate within the cavities formed by enzymes such as CelA [23]. We also note that the *C. bescii* genome does not contain a CBM2. In addition, the linker region between the GH5 domain and the CBM2 of E1 is different from those in *C. bescii* in terms of sequence homology and amino acid composition. In *C. bescii*, only one GH5 domain, the C-terminus portion of Cbes1866 (CelB), exhibits high amino acid sequence homology (98 % of sequence query cover and 35 % sequence identity) with the catalytic domain of E1. The low sequence homology of the other GH5 domains in *C. bescii* (Additional file 1: Table S1) suggests they probably contain catalytic activities differing from E1 making it an ideal candidate to supplement the GH5 containing enzymes in the *C. bescii* secretome.

There are also several potential advantages to expressing E1 in *C. bescii* to improve its performance in biomass deconstruction. Cultures can be grown at higher temperatures matching the optimum cellulolytic activities of thermostable E1 (*T*_opt_ about 81 °C) [21, 22]. E1 is active over a broad range of pH and still quite active at a low pH (~pH 5.5) [21, 22], which is similar to the optimum pH range for CelA and covers the spectrum of pH changes of media acidification during *C. bescii* growth (pH drop from pH 7.2 to ~5.0) [24]. The resistance of engineered E1 to feedback inhibition by cellobiose has been reported [25] which may be important for *C. bescii* cellulolytic activity. Moreover, no extracellular β-glucosidase has been identified in the *C. bescii* genome. While *C. bescii* does contain a β-glucosidase (Cbes0458), it does not contain an identifiable signal sequence.

Here we report synergistic activity between E1 and CelA in vitro and in vivo. The E1 gene from *A. cellulolyticus* was cloned and expressed in *C. bescii* and the secretome of the resulting strain showed increased activity on both CMC and Avicel. These data suggest that while extremely effective, the ability of the *C. bescii* secretome to deconstruct plant biomass may be improved with the addition of key enzymes.

## Results and discussion

### Synergy between E1 and CelA, the primary exoglucanase in the *C. bescii* secretome, in vitro

**Fig. 1:**
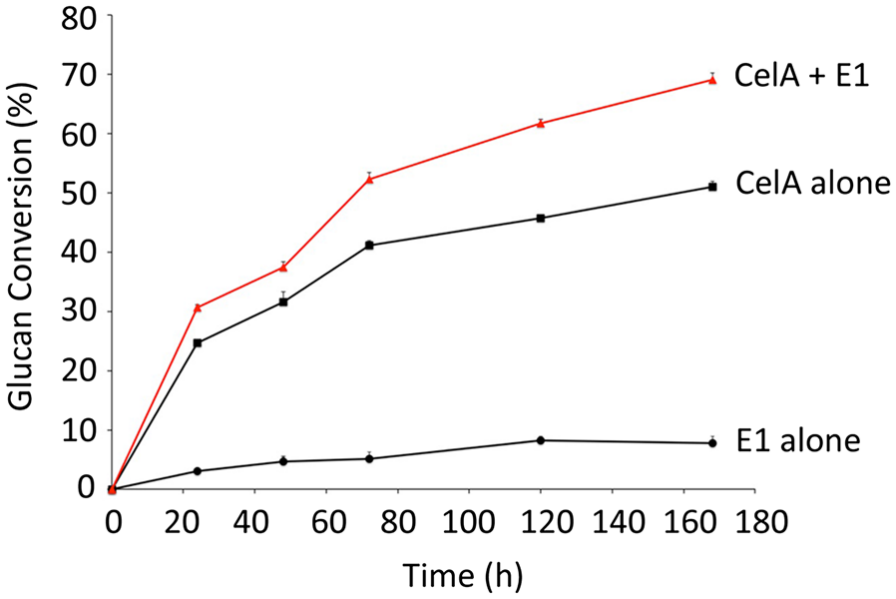
Synergistic effect of purified *C. bescii* CelA and *A. cellulolyticus* E1 enzymes in vitro. Avicel hydrolysis by *C. bescii* CelA (*black squares*) at an enzyme loading of 15 mg/g, *A. cellulolyticus* E1 (*black dots*) at an enzyme loading of 4 mg/g, and a mixture of *C. bescii* CelA and *A. cellulolyticus* E1 (*red triangles*) at an enzyme loading of 11 and 4 mg/g, respectively.

For action on crystalline cellulose, cellulase systems are typically weak in exoglucanase activity, not endoglucanase activity [26]. Endoglucanases are needed catalytically and usually account for no more than ~20 % of most cellulase secretomes. EI is more than a typical endoglucanase, because it has some processive qualities. To investigate the possibility that the *C. bescii* CelA and the *A. cellulolyticus* E1 enzymes might act synergistically in vitro, we tested both CelA and E1 separately on Avicel at an enzyme loading of 15 and 4 mg/g, respectively. In the absence of an exoglucanase, E1, converts only 8 % of the Avicel in 7 days. CelA alone converts 51 % (Fig. 1). The combination of CelA with E1 at the same total enzyme loading as CelA alone, allows the conversion of more than 69 % of the Avicel over the course of 7 days (Fig. 1). This represents an improvement of more than 10 % that can be directly attributed to synergy between the two enzymes in vitro.

### Heterologous expression and secretion of the E1 protein from *A. cellulolyticus* in *C. bescii*

**Fig. 2:**
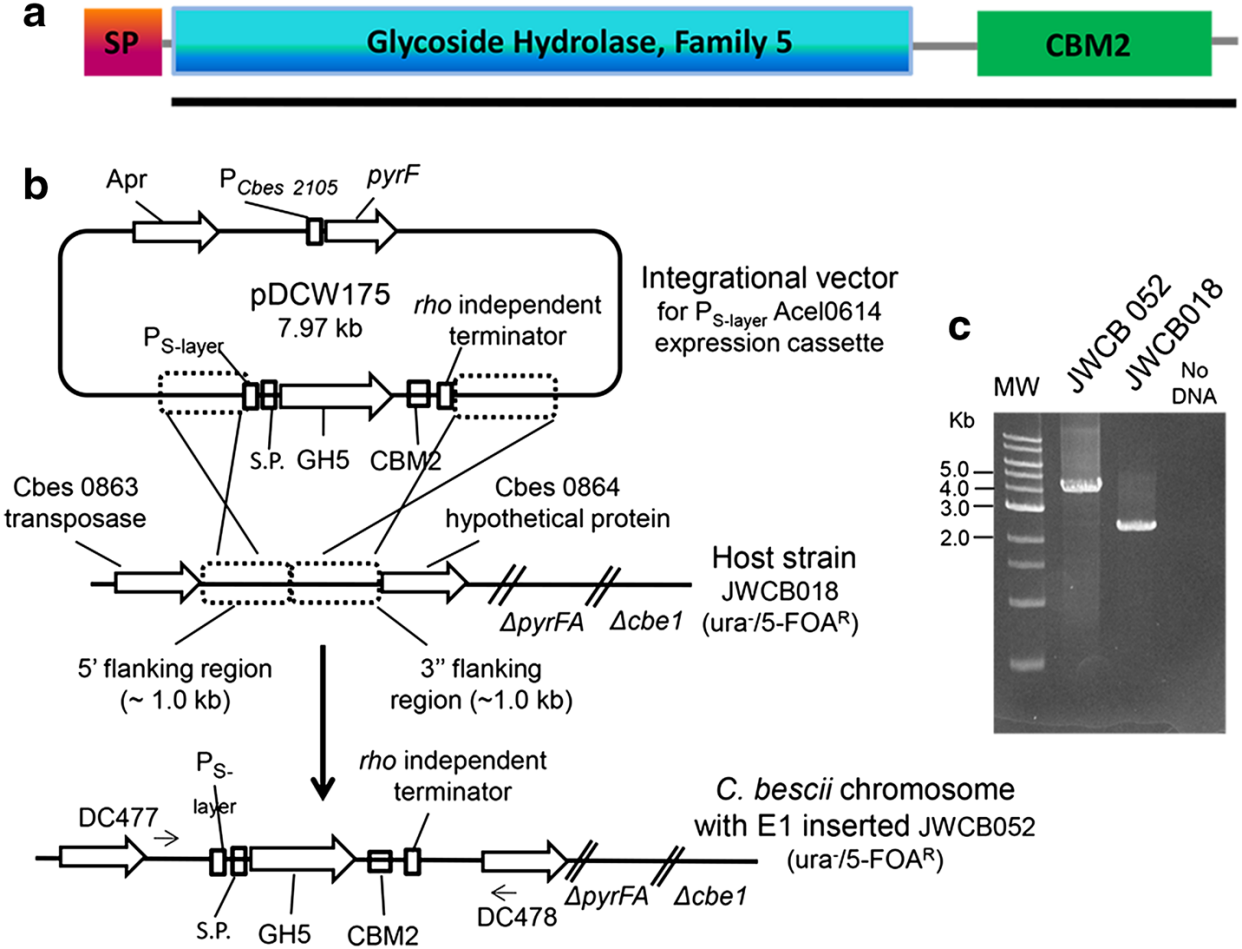
Chromosomal integration of the E1 gene into the *C. bescii* genome. **a** A diagram of native E1 protein: SP, signal peptide; a family 5 endoglucanase; and CBM2, a family 2 carbohydrate-binding module/domain. The *black bar* beneath the diagram represents the portion of the pDCW175 construct derived from Acel_0614. **b** A depiction of the chromosomal location and integration event of the E1 expression cassette/secretion cassette. **c** Agarose gel showing PCR products amplified using primers DC477 and DC478 annealing to regions outside the site of integration in the newly constructed strain JWCB052 *ΔpyrFA ldh::ISCbe4 Δcbe1::*P_S-layer_ *acel0614 E1* containing the E1 expression cassette, 4.079 kb (*lane 2*) and the parent strain, JWCB018 *ΔpyrFA* *ldh::ISCbe4 Δcbe1*, 2.440 kb (*lane 3*); DNA MW standards (*lane 1*); no template PCR control (*lane 4*).

To express full length E1 protein (Acel 0614) from *A. cellulolyticus* (Fig. 2a) in *C. bescii*, the gene was amplified by PCR from *A. cellulolyticus* chromosomal DNA and cloned into an integrational expression vector, pDCW175 (Fig. 2b, Additional file 1: Figure S1) under the transcriptional control of the *C. bescii* S-layer promoter [27]. The *C. bescii* CelA signal peptide sequence was added upstream of the GH5 domain of the E1 protein, and a C-terminal histidine-tag was added to facilitate future protein purification. The signal peptide derived from the *C. bescii celA* gene was used for secretion of E1. CelA is the most abundant extracellular protein produced by *C. bescii* [11, 19], suggesting that its signal sequence works well. We recently demonstrated the use of this signal peptide for the expression of full length CelA in *C. bescii* [28]. The native E1 signal sequence shows no sequence homology with any signal peptides in *C. bescii*. The CelA signal sequence was fused to the 5′ end of the coding sequence of the E1 catalytic domain, replacing the E1 signal peptide, to create the E1 expression/secretion cassette in pDCW175.

This plasmid, that contained a wild-type copy of the *C. bescii pyrF* gene, was transformed into JWCB018, a strain containing a deletion of *pyrF* resulting in uracil auxotrophy, and transformants were selected for uracil prototrophy (Fig. 2b; Table 1) [29, 30]. JWCB018 also contains a deletion of *Cbe*I, a restriction endonuclease, to facilitate transformation of DNA from *E. coli* [30, 31]. The plasmid was targeted to integrate into an inter-cistronic region on the *C. bescii* genome previously determined to be available without affecting growth or resulting in a detectable phenotype [27]. Transformants containing an integrated vector were then treated with 5-FOA to select for excision of the plasmid sequences leaving the EI expression cassette in the chromosome. Integration of the plasmid into the targeted site in the *C. bescii* chromosome as well as the presence of the P_*S*-*layer*_*acel0614* (*E1)* cassette after strain construction was confirmed by PCR amplification of the relevant chromosome regions (Fig. 2c) as well as sequencing of the PCR products. The resulting strain JWCB052 (*ΔpyrFA ldh::ISCbe4 Δcbe1* P_*S*-*layer*_E1) was used for further analysis.

**Table 1:** Strains and plasmids used in this work

Strains/plasmids	Strain and genotype/phenotype	References
*C. bescii*		
JWCB001	Wild type (*ura* ^+^/5-FOA^S^)	DSMZ^a^
JWCB018	*ΔpyrFA ldh::ISCbe4 Δcbe1* (*ura* ^−^/5-FOA^R^)	[29]
JWCB052	*ΔpyrFA ldh::ISCbe4 Δcbe1::*P_S-layer_ *acel0614 (E1)* ^b^ (*ura* ^-^/5-FOA^R^)	This study
*Escherichia coli*		
JW314	DH5α containing pDCW144 (Apramycin^R^)	[27]
JW336	DH5α containing pDCW174 (Apramycin^R^)	This study
JW337	DH5α containing pDCW175 (Apramycin^R^)	This study
Plasmids		
pDCW144	Integrational vector for *Caldicellulosiruptor bescii* (Apramycin^R^)	[27]
pDCW174	Integrational vector containing signal peptide of CelA for *Caldicellulosiruptor bescii* (Apramycin^R^)	This study
pDCW175	Integrational vector containing the P_S-layer_ *acel0614 (E1)* ^b^ expression cassette (Apramycin^R^)	This study

^a^ *German Collection of Microorganisms and Cell Cultures*.

^b^ *acel0614 (E1)* [glycoside hydrolase (family 5) with cellulose-binding (family 2) derived from *Acidothermus cellulolyticus 11B*].

**Fig. 3:**
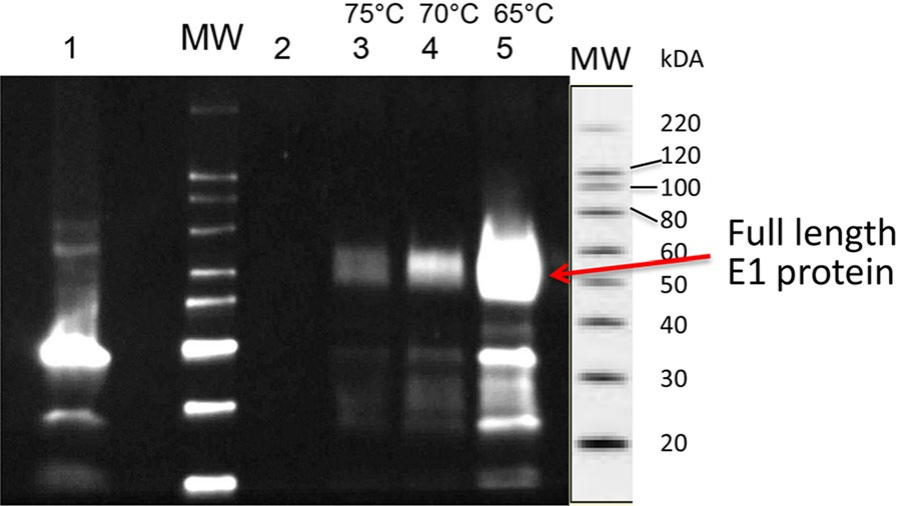
Confirmation of E1 expression and secretion in *C. bescii* using Western blot analysis. Concentrated extracellular proteins (10 µg) were electrophoresed in a 15 % gradient Mini-Protean TGX gel (BIO-RAD) and electro-transferred to a PVDF membrane (ImmobilonTM-P; Millipore). The membrane was then probed with an E1 monoclonal antibody. Truncated version of E1 produced in *Streptomyces lividans* (*lane 1*); parent strain JWCB018 *ΔpyrFA* *ldh::ISCbe4 Δcbe1* grown at 75 °C (*lane 2*); E1 expression strain JWCB052 *ΔpyrFA* *ldh::ISCbe4 Δcbe1::P*_*S*-*layer*_ *acel0614 (E1)* grown at 75 °C (*lane 3*), 70 °C (*lane 4*), and 65 °C (*lane 5*); MW, MagicMark™ molecular weight marker (Invitrogen).

Expression and secretion of E1 by *C. bescii* was confirmed by Western blot analysis using a mouse anti-E1 monoclonal antibody to detect the EI protein from the concentrated supernatant of a verified transformant (JWCB052). Concentrated culture supernatants from the E1 transformant (JWCB052) grown at varying temperatures (65, 70, and 75 °C) were electrophoresed on a 4–15 % gradient Mini-Protean TGX gel (BIO-RAD) and transferred to a PVDF membrane and developed with the E1 antibody. The positive control for detection of E1 protein was a truncated fragment of the E1 catalytic domain (40 kDa) produced in *Streptomyces lividans* (Fig. 3). A ~60 kDa band was visible in the culture supernatants corresponding to the predicted size for full length E1 protein (Fig. 3). These data confirm expression and extracellular localization of E1 in *C. bescii*.

**Fig. 4:**
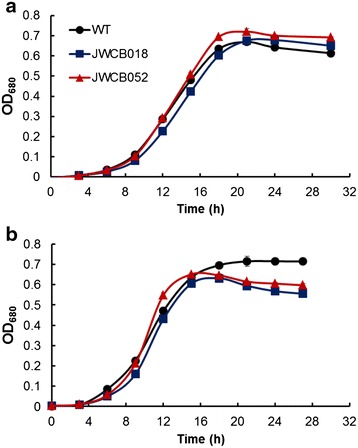
Growth of the wild type, parent strain (JWCB018), and E1 expression strain (JWCB052) on cellobiose. Growth of the wild type (*black circles*); JWCB018, *ΔpyrFA* *ldh::ISCbe4 Δcbe1* (*blue squares*); and JWCB052, *ΔpyrFA* *ldh::ISCbe4 Δcbe1::P*_*S*-*layer*_ *acel0614 (E1)* (*red triangles*) strains on cellobiose. **a** Growth at 65 °C and **b** growth at 75 °C.

As shown in Fig. 3, the most abundant amount of extracellular E1 protein was detected in cells grown at 65 °C, with lesser amounts from cells grown at either 70 or 75 °C. E1 originates from *A. cellulolyticus*, which has an optimum growth temperature of 55 °C, whereas *C. bescii* has an optimum growth temperature of 78 °C. There are a number of issues with the heterologous expression and secretion of proteins from mesophilic sources in thermophiles, like *C. bescii*. In addition to messenger RNA instability and post-translational proteolysis by host proteases, protein stability at high temperature is often a problem. In addition, there is severe codon usage bias due to differences in the GC content of the E1 gene (61 %) and the *C. bescii* genome (35 %) [32]. This bias can affect E1 expression due to limitations in supply of certain tRNA species. Insufficient tRNA pools can lead to translational stalling and premature termination. We observed a ~30 % increase in doubling time at 65 °C (2.3 h) compared to 75 °C (1.6 h) (Fig. 4). Slower growth might help overcome the codon usage bias by slowing down transcription and translation rates to allow the incorporation of rare tRNAs which corresponds to our observed increase in protein at the lower temperature (Fig. 3).

The E1 endoglucanase has limited activity on the disaccharide, cellobiose [33], so to test whether or not expression of E1 in *C. bescii* resulted in a growth defect, growth of the wild type, the parent strain, and the E1 producing strain on cellobiose were compared. To examine the overall effect of E1 production and secretion on the growth of *C. bescii*, growth on cellobiose was monitored by OD_680_ at 65 and 75 °C for the E1 producing strain (JWCB052), the parent strain (JWCB018), and the wild-type strain (Fig. 4). Expression of E1 in *C. bescii* had no detectable effect on the growth of cells at either 65 or 75 °C (Fig. 4).

### Expression of E1 in *C. bescii* results in enhanced activity of extracellular fractions on both Avicel and carboxymethylcellulose

**Fig. 5:**
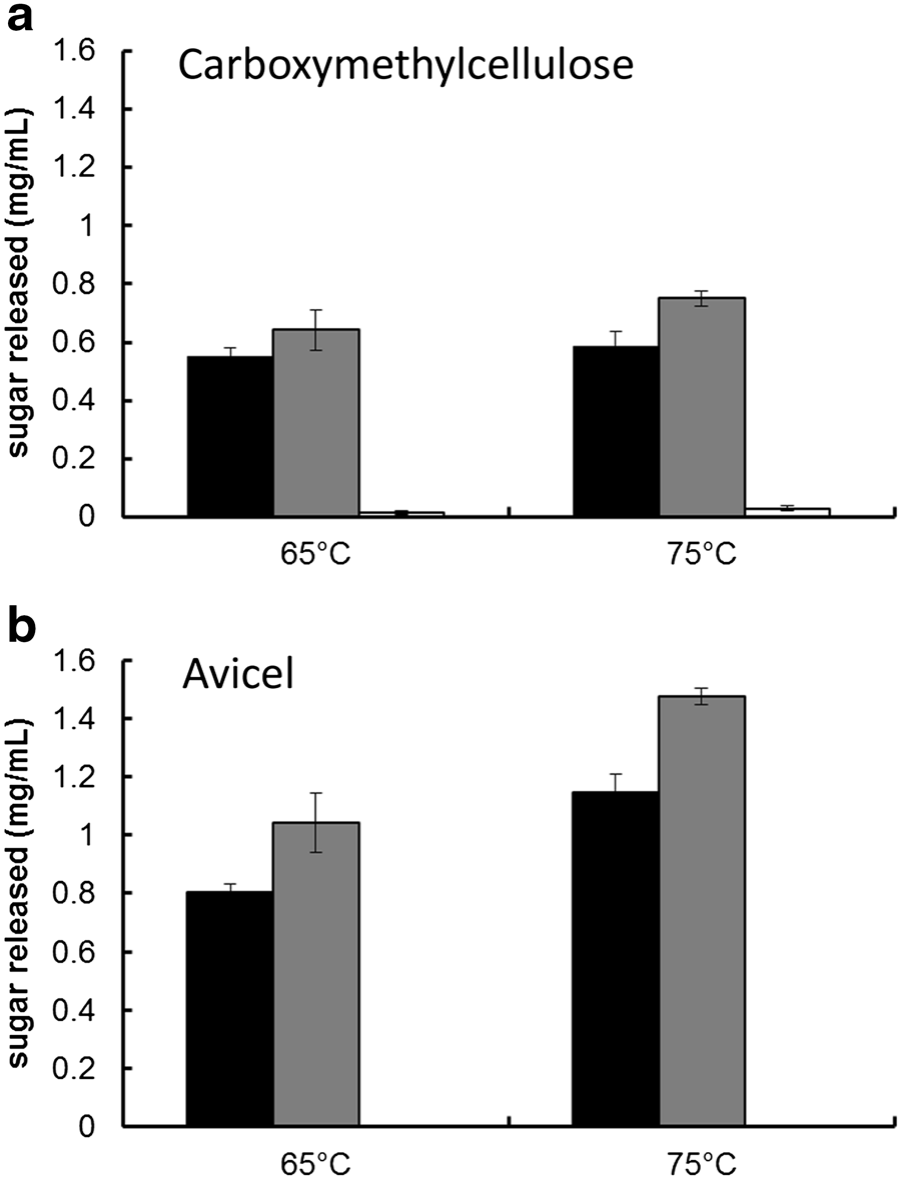
Relative quantification of enzymatic activity of the extracellular fraction of *C. bescii* expressing E1 (Acel0614) on Avicel and carboxymethylcellulose. Carboxymethylcellulose (CMC) or Avicel was used as substrate at either 65 or 75 °C. **a** Activity of the extracellular fraction (25 µg/mL concentrated protein) on CMC from the parent strain JWCB018 *ΔpyrFA* *ldh::ISCbe4 Δcbe1* (*black*), the E1 expression strain, JWCB052 *ΔpyrFA* *ldh::ISCbe4 Δcbe1::P*_*S*-*layer*_ *acel0614 (E1)* (*grey*), and no enzyme control (*white*). **b** Activity of the extracellular fraction (25 µg/mL of concentrated protein) on Avicel from the parent strain JWCB018 (*black*), the E1 expression strain, JWCB052 (*grey*), and no enzyme control (*white*).

To test if expression of E1 in *C. bescii* affected the cellulolytic activity of the extracellular enzyme fraction, enzyme assays were performed from concentrated culture supernatants of *C. bescii* cells producing E1 protein (JWCB052) compared to the *C. bescii* parent strain (JWCB018). Cells were grown at 65 °C as E1 was produced at the highest levels at that temperature (Fig. 3). Even though *A. cellulolyticus* grows optimally at the temperature of the hot spring from which it was collected, 55 °C [34], the E1 protein has been shown to be active at considerably higher temperatures, with an optimum of 81 °C (in vitro) [21, 22, 35]. Supernatants were then concentrated and exchanged with buffer before assaying on CMC for 1 h or on Avicel for 24 h at 65 and 75 °C. CMC is traditionally used as an assay for endoglucanase activity while Avicel is used to assay exoglucanase activity [36]. Increased activity of concentrated culture supernatants from the E1 expression strain (JWCB052) was observed on both CMC and Avicel, and at both assay temperatures (65 and 75 °C) compared to the parent strain (JWCB018). These data are based on two independent biological replicates (Fig. 5, Additional file 1: Figure S2). Activity on Avicel increased on average 17.5 % at 65 °C and 16.4 % at 75 °C (Fig. 5b, Additional file 1: Figure S2B), while activity on CMC increased on average 10.8 % at 65 °C and 12.6 % at 75 °C (Fig. 5a, Additional file 1: Figure S2A). The fact that there is a larger increase in exoglucanase activity (on Avicel) in the E1 containing strain than endoglucanase activity (on CMC) suggests that the addition of the E1 endoglucanase results in the generation of increased chain ends to improve cellulolytic activity. We conclude from these data that the endoglucanase activity of the *C. bescii* secretome, and likely CelA, is limiting in its activity on cellulose.

## Conclusions

The E1 and CelA enzymes act synergistically in vitro to digest Avicel despite the fact that CelA contains an endoglucanase activity, albeit a tethered one. This suggests that the ability to generate new cellulose chain ends may be rate limiting in cellulose hydrolysis and that the addition of the smaller E1 augments this activity resulting in increased activity. The increase in activity observed when E1 is added to the secretome of *C. bescii* is very promising. Despite an abundance of cellulolytic enzymes, including several endoglucanases in *C. bescii*, the addition of E1 showed a measurable difference in cellulolytic activity. Further modifications could lead to improvements for *C. bescii* as both a microorganism for consolidated bioprocessing and as an expression host for secretion of heterologous proteins especially those originating from thermophiles. The increase in protein yields at lower growth temperatures suggests that codon usage bias is likely an issue for expression of E1 in *C. bescii*. Codon optimization might well improve expression at higher temperatures and increase cellulolytic activity on biomass substrates. Opportunities also remain for improving the E1 protein itself. Linker regions within multi-domain enzymes like CelA have been implicated in protein stability, glycosylation, and flexibility for enzymatic activity. The linker region of E1 shows no homology to those found in the multi-domain proteins of *C. bescii* and swapping the linker region of E1 with ones found in CelA, for example, might increase its activity. Likewise, since there is no homology between the CBM2 of E1 with those in *C. bescii*, swapping the CBM domains of *C. bescii* cellulases with the CBM2 of E1 might improve the substrate binding versatility of these enzymes. This work also offers the possibility of constructing hybrid proteins by gene fusions between various GH domains and CBMs, such as those found in CelA to produce new multi-domain enzymes with novel catalytic activity.

## Methods

### Strains, media and culture conditions

*Caldicellulosiruptor bescii* strains, *E. coli* strains, and plasmids used in this study are listed in Table 1. *C. bescii* strains were grown anaerobically in liquid or on solid surface in low osmolarity defined (LOD) medium [24] with maltose (0.5 % wt/v; catalog no. M5895, Sigma) as the carbon source, final pH 6.8, at 75 °C as previously described [29, 30]. Liquid cultures were grown from a 0.5 % inoculum or a single colony and incubated at 75 °C in anaerobic culture bottles degassed with five cycles of vacuum and argon. For uracil auxotrophs, JWCB018 and JWCB052, the LOD medium was supplemented with 40 μM uracil. *E. coli* strain DH5α was used for plasmid DNA constructions and preparations. Techniques for *E. coli* were performed as described [37]. *E. coli* cells were grown in LB broth supplemented with apramycin (50 μg/mL) and plasmid DNA was isolated using a Qiagen Mini-prep Kit. Chromosomal DNA from *C. bescii* strains was extracted using the Quick-gDNA™ MiniPrep (Zymo) or using the DNeasy Blood & Tissue Kit (Qiagen) according to the manufacturer’s instructions. *E. coli* strain DH5α cells were transformed by electroporation in a 2-mm-gap cuvette at 2.5 V and transformants were selected for apramycin resistance.

### Vector construction for the knock-in of Acel_0614 into *C. bescii*

The plasmids described below were generated using Q5 High-Fidelity DNA polymerase (New England BioLabs) for PCR reactions, restriction enzymes (New England BioLabs), and the Fast-link DNA Ligase kit (Epicentre Biotechnologies) according to the manufacturer’s instructions. Plasmid pDCW174 (Table 1) was constructed by inserting the sequence of the CelA (Cbes1867) signal peptide into pDCW144 [27], which also contains the regulatory region of Cbes2303 and encodes a *C*-terminal 6X histidine-tag and a *rho*-independent transcription terminator. The 6.3 kb DNA fragment was amplified with primers DC464 (adding a *Bam*HI site) and DC466 (adding an *Sph*I site) using pDCW144 as template. A 1.752 kb DNA fragment containing the coding sequence of the N-terminus portion (including the 72 bp signal peptide sequence) of Cbes1867 was amplified with DC368 (with *Sph*I site) and DC560 (with *Bam*HI site) using *C. bescii* gDNA as a template. These two linear DNA fragments were digested with *Bam*HI and *Sph*I, and then ligated to construct pDCW174 (8.05 kb) (Table 1). The 6.41 kb DNA fragment, containing the Cbes 2303 regulatory region, the 72 bp signal peptide sequence, a *C*-terminal 6X histidine-tag and the rho-independent transcription terminator, was amplified from pDCW174 using primers DC579 (adding an *Apa*I site) and DC580 (adding a *Xma*I site) and was used as a back-bone fragment to construct pDCW175 (Fig. 2b, Additional file 1: Figure S1; Table 1). A 1.6 kb DNA fragment containing the coding sequence of Acel_0614 was amplified with DC581 (adding an *ApaI* site) and DC582 (adding an *Xma*I site) using *A. cellulolyticus 11B* gDNA as template. These two linear DNA fragments were digested with *Apa*I and *Xma*I, and then ligated to construct pDCW175 (7.97 kb) (Additional file 1: Figure S1). The DNA sequences of the primers are shown in Additional file 1: Table S2. The sequences of pDCW174 and pDCW175 were verified by Automatic sequencing (Macrogen USA, Maryland). Plasmids are available upon request.

### Transformation, screening, purification, and sequence verification of engineered *C. bescii*

To construct strain JWCB052, one microgram of pDCW175 DNA was used for electroporation into JWCB018 (*ΔpyrFA ldh*::IS*Cbe4 ΔcbeI*) as previously described [29, 30]. Cells were then plated onto solid LOD medium and uracil prototrophic transformants were inoculated into liquid medium for genomic DNA extraction and subsequent PCR screening of the targeted region of the chromosome. Confirmed transformants were inoculated into nonselective liquid defined medium, with 40 μM uracil, and incubated overnight at 75 °C to allow loop-out of the plasmid. The cultures were then plated onto 5-FOA (8 mM) containing solid medium. Transformants containing the knock-in were further purified by two additional passages under selection on solid medium and screened a second time by PCR. The insertion of the Acel_0614 expression cassette at the targeted chromosome region was verified by PCR amplification and sequence analysis using primers DC462 and DC463. A PCR product was generated from genomic DNA using primers (DC477 and DC478) outside the homologous regions used to construct the knock-in. Primers and sequences used in this study are listed in Additional file 1: Table S2.

### Preparation of extracellular fractions and Western blotting

Extracellular protein (ECP) was collected from 2 L of culture grown at a range of temperatures (65, 70, or 75 °C) in closed bottles shaking at 150 rpm to an OD_680_ of 0.25–0.3. LOD media was supplemented with 40 μM uracil and 40 mM MOPS. Cultures were centrifuged (6,000×*g* at 4 °C for 15 min), filtered (glass fiber, 0.7 µm) to separate out cells, and concentrated with a 3 kDa molecular weight cut-off column (Hollow Fiber Cartridge, GE Healthcare). Protein concentrations were determined using the Bio-Rad protein assay kit with bovine serum albumin (BSA) as the standard. ECP samples (10 µg) were electrophoresed in 4–15 % gradient Mini-Protean TGX gels (BIO-RAD) for either Coomassie blue-staining or for electrotransfer to PVDF membranes (ImmobilonTM-P; Millipore) using a Bio-Rad Mini-Protean 3 electrophoretic apparatus. The membrane was probed with E1 monoclonal antibody (1:1,500 dilution, provided by Bill Adney and Steve Decker, NREL) using the ECL Western Blotting substrate Kit (Thermo Scientific) as specified by the manufacturer.

### Enzyme activity assays

Enzymatic assays using pure enzyme preparations of CelA and E1, their isolation and purification were conducted as previously described [23], were performed in 20 mM acetate buffer pH 5.5 with 100 mM NaCl and 10 mM CaCl_2_. The enzyme loadings were 15 mg/g for CelA, 4 mg/g for E1, and 11 and 4 mg/g for CelA and E1, respectively, in the mixture. Avicel digestion experiments were conducted for 7 days with constant mixing at 75 °C with sampling at various time points. Enzymes were inactivated by boiling for 15 min after which samples were filtered through 0.45 µm Acrodisc syringe filters. The released sugars were analyzed by HPLC. Samples were injected at 20 mL and run on an Agilent 1100 HPLC system equipped with a BioRad Aminex HPX-87H 300 mm × 7.8 mm column heated to 55 °C. A constant flow of 0.6 mL/min was used with 0.1 M H_2_SO_4_ in water as the mobile phase to give optimal sugar separation. Glucose and cellobiose were quantified against independent standard curves and converted to anhydrous glucan equivalent and the results are reported as anhydrous glucan converted. All experiments were performed in triplicate and the resulting extents of conversion are shown as percent glucan converted.

To assess the activity of the secretome of the *C. bescii* strain containing E1, concentrated extracellular protein (ECP) was buffer exchanged with 20 mM MES/2 mM β-mercaptoethanol (pH 5.5). Protein concentrations were determined using the Bio-Rad protein assay kit with bovine serum albumin (BSA) as the standard. Cellulolytic activity was determined using 10 g/L of either CMC or Avicel in MES reaction buffer (pH 5.5) as previously described [38]. Twenty five microgram per milliliter of extracellular protein was added to each reaction and incubated at 65 or 75 °C (1 h for CMC and 24 h for Avicel). Controls were incubated for the same length of time without added enzyme. Reducing sugars in the supernatant were measured using dinitrosalicylic acid (DNS). Samples and standards (glucose) were mixed 1:1 with DNS and boiled for 2 min and measured at OD_575_. Activity was reported as mg/mL of sugar released.

## Additional file

Additional file 1:
**Figure S1.** Plasmid map of chromosomal knock-in vector in *C. bescii* for extracellular expression of E1 (Acel0614). **Figure S2.** Relative quantification of enzymatic activity of the extracellular fraction of *C. bescii* expressing E1 (Acel0614) on Avicel and carboxymethylcellulose. **Table S1.** The list of Glycoside Hydrolase Family 5 (GH5) catalytic domains in *Caldicellulosiruptor bescii* and their sequence homology with the GH5 domain in E1 from *A. cellulolyticus.* **Table S2.** Primers used in this study.

## Abbreviations

*C. bescii*: *Caldicellulosiruptor bescii*
*A. cellulolyticus*: *Acidothermus cellulolyticus*
CBM: carbohydrate-binding module
GH: glycoside hydrolase
CAZy: carbohydrate-active enzymes
CMC: carboxymethylcellulose
YE: yeast extract
5-FOA: 5-fluoroorotic acid
LOD: low osmolarity defined
LB broth: Luria Bertani broth
ECP: extracellular protein
BSA: bovine serum albumin
DNS: dinitrosalicylic acid
