# Supplementary material for: Expression of the Acidothermus cellulolyticus E1 endoglucanase in Caldicellulosiruptor bescii enhances its ability to deconstruct crystalline cellulose
Source: Biotechnol Biofuels. 2015 Aug 13;8:113. doi: 10.1186/s13068-015-0296-x (PMC4533959; doi:10.1186/s13068-015-0296-x)
Supplement: Additional file 1: — Figure S1. Plasmid map of chromosomal knock-in vector in C. bescii for extracellular expression of E1 (Acel0614). Figure S2. Relative quantification of enzymatic activity of the extracellular fraction of C. bescii expressing E1 (Acel0614) on Avicel and carboxymethylcellulose. Table S1. The list of Glycoside Hydrolase Family 5 (GH5) catalytic domains in Caldicellulosiruptor bescii and their sequence homology with the GH5 domain in E1 from A. cellulolyticus. Table S2. Primers used in this study. [file 13068_2015_296_MOESM1_ESM.docx]

**Supplementary materials**


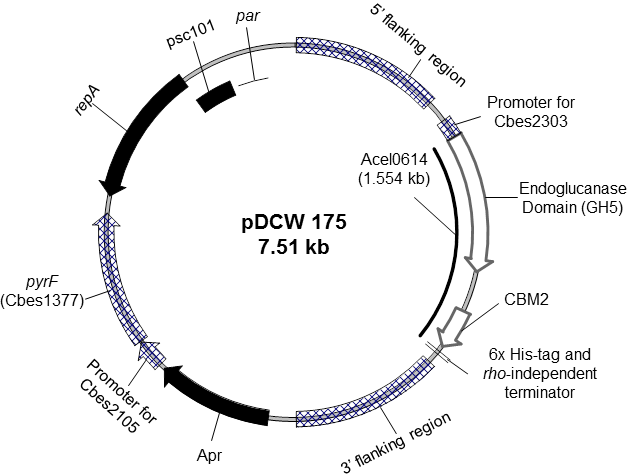


**Fig. S1. Plasmid map of chromosomal knock-in vector in *C. bescii* for extracellular expression of E1 (Acel0614).** An expression cassette, which contains the regulatory region of the *C. bescii* S-layer protein (Cbes2303), a *C*-terminal 6X His-tag version of *Acel0614*, and a *rho*-independent terminator, is shown. The cross-hatched boxes represent the sequences originated from *C. bescii*. The black arrows and box represent the sequences derived from *E. coli*. The apramycin resistant gene cassette (Apr); *psc101,* low copy replication origin in *E. coli*; *repA*, a plasmid-encoded gene required for *psc101* replication; *par*, partition locus; *pyrF* cassette; 5’ and 3’ franking sequences for homologous recombination into the targeted insertion site in *C. bescii* chromosome are indicated.


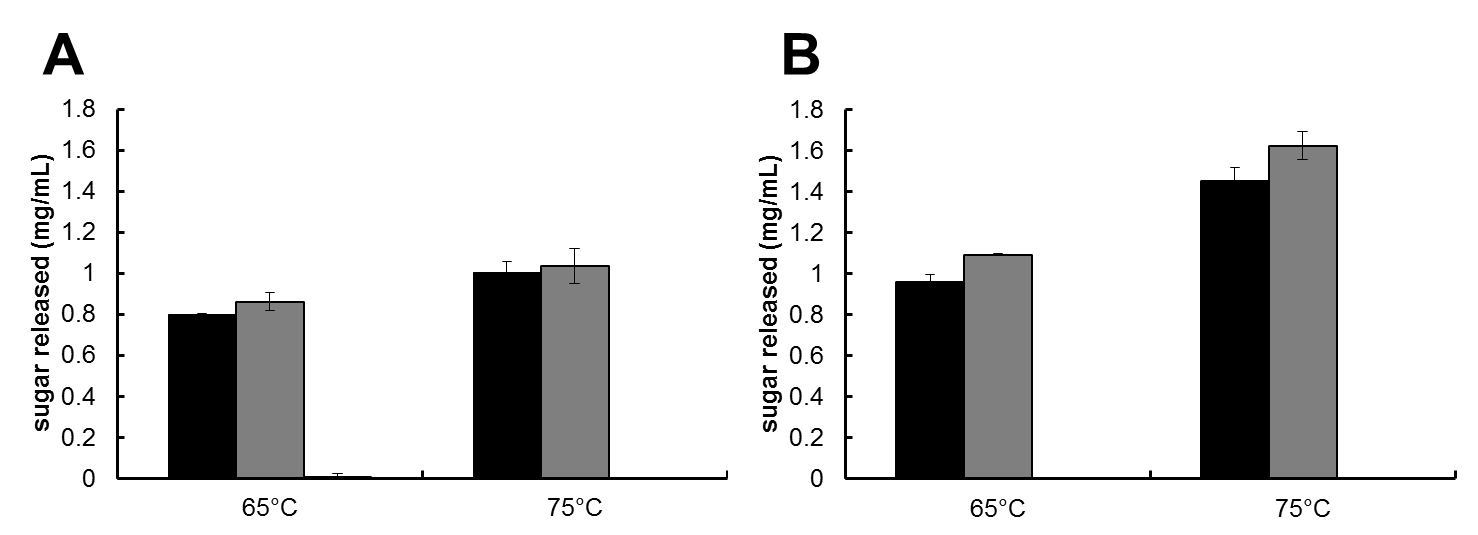


**Fig. S2. Relative quantification of enzymatic activity of the extracellular fraction of *C. bescii* expressing E1 (Acel0614) on Avicel and carboxymethylcellulose.**

Carboxymethylcellulose (CMC) or Avicel was used as substrate at either 65°C or 75°C. A) Activity of extracellular protein (25 µg/mL concentrated protein) against CMC from the parent strain JWCB018 (*ΔpyrFA* *ldh::ISCbe4 Δcbe1*) (black), the E1 expression strain, JWCB052 (*ΔpyrFA* *ldh::ISCbe4 Δcbe1::P_S-layer_ acel0614 (E1))* (grey), and no enzyme control (white). B) Activity of extracellular protein (25 µg/mL of concentrated protein) against Avicel from the parent strain JWCB018 (black), the E1 expression strain, JWCB052 (grey), and no enzyme control (white).

**Table S1**. The list of Glycoside Hydrolase Family 5 (GH5) catalytic domains in *Caldicellulosiruptor bescii* and their sequence homology with the GH5 domain in E1 from *A. cellulolyticus.*

| Gene | Predicted gene product | Carbohydrate-Active enzymes (CAZy) module architecture | GH5 Sequence query coverage/Sequence identity |
| --- | --- | --- | --- |
| Cbes0234 | Cellulase (glycoside hydrolase family 5) | GH5 | 61%/26% |
| Cbes0594  (CelD) | endo-β-1,4-glucanase D | GH5-CBM28 | 23%/25% |
| Cbes1859 (CbMan5A/Cel44A) | bifunctional mannanase/ endoglucanase | GH5-CBM3-CBM3-GH44 | 38%/22% |
| Cbes1865 (CelC-ManB) | bifunctional cellulase/β-mannanase | GH9-CBM3-CBM3-CBM3-GH5 | 38%/22% |
| Cbes1866 (CelB;CbMan5C/Cel5A) | bifunctional mannanase/  endoglucanase | GH5(a)-CBM3-CBM3-CBM3-GH5(b) | a: 38%/22%  b: 98%/35% |

**Table S2.** Primers used in this study.

| Primers | Sequences (5’ to 3’) | Description |
| --- | --- | --- |
| DC368 | AGAGCATGCTGTGCCAGTAAAGTCTACTAAAATGTAG | To construct pDCW174 |
| DC462 | TGCTGGCAGAGAAGAGCGAAA | Sequencing primer for pDCW174 and 175 |
| DC463 | TCTTCATCCCAATCTTCAACTTC | Sequencing primer for pDCW174 and 175 |
| DC464 | ACTGGATCCCTCACCAAACCTCCTTGTATGAT | To construct pDCW174 |
| DC466 | AGAGCATGCCATCACCATCACCATCACTAATAATAAAGC TGAAATAAAAGAGGGTGAGA | To construct pDCW174 |
| DC477 | TGGTTGACCAGGAGAATTTTACACA | To verify the targeted insertion of P_S-layer_Acel_0614 |
| DC478 | AGCAACAATCCTGCATTTGTAAG | To verify the targeted insertion of P_S-layer_Acel_0614 |
| DC560 | AGAGGATCCATGAAGCGTTACAGAAGAATTATTGCCA | To construct pDCW174 |
| DC579 | ACTGGGCCCAAACGAACCAGCCCTAACCTCT | To construct pDCW175 |
| DC580 | ACACCCGGGCATCACCATCACCATCACTAATAAT | To construct pDCW175 |
| DC581 | ACAGGGCCCTATTGGCACACGAGCGGC | To construct pDCW175 |
| DC582 | ACACCCGGGACTTGCTGCGCAGGCGACT | To construct pDCW175 |
| DC584 | ATCCCCAGTTCTTGTTCCAG | Sequencing primer for pDCW175 and P_S-layer_Acel_0614 |
| DC585 | TCATTTTCGTCGAAGGTGTG | Sequencing primer for pDCW175 and P_S-layer_Acel_0614 |
